# Treatment of Pain

**Published:** 1888-05

**Authors:** James Maughan


### AMERICAN JOURNAL

—OF—

### DENTAL SCIENCE.

VOL. XXII. THIRD SERIES—MAY, 1888. NO. 1.

ARTICLE I.

### TREATMENT OF PAIN.

BY JAMES MAUGHAN, M. D., L. R. C. P., M. R. C. S.

[A paper read before the Students' Society of the National Dental College.

*Mr. President and Gentlemen:*—The subject I have chosen for discussion is one that will commend itself at once to the humanitarian and the scientific instincts of all here to-night. In putting my thoughts together, I shall adhere as nearly as I can to the dental aspect of the question, and so invite your kindly criticism.

The treatment of pain must be premised by a word or two as to its pathology. What has baffled the sorcerers and philosophers of old still baffles the neurologists of to-day, and they are unable to state clearly in so many words, or to demonstrate practically to sufferers or non-sufferers, the true pathology of pain. That there is an invisible and subtle something passing along sensory nerves, and that the consciousness must be in unbroken connection with such nerves are hypotheses that all must admit. And, moreover, whatever the impalpable something may be in its essence, we can go one step further, and assert that it is *always* associated with degenerative changes and lowered vitality in the nerve itself. If we cannot formulate in exact terms the real nature of pain, we are equally wanting in an instrument by which we can perceive its presence and measure its intensity.

Gentlemen, an immortal name awaits the one among you who will invent an æsthesiometer and give to the world a means of detecting instantly the tricks of a malingerer, or of diagnosing a case of hysteria rapidly and easily. In byegone days a man was bled because he was living at too fast a rate; his chest was cupped for a disease exhausting enough in itself. Aconite and antimony, both powerful depressants, to say nothing of mercury with its hydra-headed sequelæ; were then pretty active in the market. The touch-stone of the pathology of the day was that organs at times took on an over-action or excess of function, and the recognised and orthodox treatment was to check such excess.

Mark the logic and pathology in a given case: a young woman, pale and whose features are blunted by an anæmic dropsy, came to me this morning complaining of her heart beating. According to our forefathers, the heart acting too quickly requires some sedative to slow its pulsation. Such a line of treatment would be a fatal failure. The blood is very scantily furnished with red corpuscles, every organ in the body is supplied with insufficient nutriment; the heart does its duty in pumping the blood to the medulla oblongata, but the blood it pumps is so poor that the centre for the vagus nerve acts but feebly; the heart thus loses the advantage of its controlling action, and, in short, gallops away under the cruel spur of the sympathetic. The right treatment, undoubtedly, is to improve the blood with the perchloride of iron and general tonic treatment. We must learn to regard these alleged over-action, then, with a sceptic's eye, and seek for the real cause of suffering in something beyond.

This cause is invariably present; it is our duty to discover it and our prerogative to remove it. Toothache, then is not an over-acting dental nerve, it is not a spontaneous hyperæsthesia suddenly and facetiously developed; it is the evidence that some definite irritation exists either in the neighborhood or in some remote spot. You will readily understand that for one and the same lesion the degree of pain varies according to the individual sensitiveness of the cortex of the brain or the tone and vigor of the nerve affected. And again, the neuralgia of a big nerve is far more severe than that of a small one. You may remember the dogma of Romberg that pain in a nerve is its prayer for purer blood. But nerves are not so intellectual; they are more like babies, they cry out when they are hurt. Now it requires some pains and patience to find out where they are hurt, but as surely as the cause is sought for with a clear head and an impartial eye, so surely will it be discovered. If we make up our minds to discountenance the strictly symptomatic form of treatment as opposed to the thoughtful and rational, we will make fewer mistakes and better men. It is no easy task to face the thousand and one variations and mimicries the nervous system plays to us, and to be able to read the riddles aright. Still, if we do our clinical work carefully and conscientiously, we will discover the best treatment, though it may be in an empirical way.

The situation of pain you usually meet with is where the sensory filaments of the fifth cranial nerve are distributed. Now, this nerve, as you know, has three branches; that is to say, the ophthalmic, the superior maxillary, and the inferior maxillary.

Ophthalmic neuralgia is far and away the most common and expresses itself most acutely in five foci, that at the supraorbital foramen being the most frequently affected.

But pain occurring in one supraorbital area, at periodic intervals between the attacks, is usually due to malaria, and a pain felt by the supraorbital nerve on the vertex of the head, as if a nail or spike were being driven in, is a peculiarly hysterical symptom. Syphilis, gout, and rheumatism have a great partiality for fibrous structures, and for causing a thickening thereof, Now such a thickening just at the opening of a bony canal, whence a nerve emerges, may press very prejudically upon that nerve, impede its nutrition, and cause severe neuralgia. Imagine such a state of things at the supraorbital foramen.

I mention these incidents and cases that you may be on the alert not to come to a conclusion too rashly, and condemn a tooth to a summary execution without due deliberation. For sometimes your patient may be the very tempter himself to lead you astray from the path I would have you walk in. Take a case in point. A man comes to you complaining of a certain tooth, and details the agonies and sleepless nights it has caused him. You listen attentively, nay, sympathetically. On examining the tooth he indicates, you find it to be perfectly sound. What is your bounden duty? Just examine the teeth in the neighbourhood, and then you may find a tooth or its periosteum gone wrong. Questioning your patient further, you learn that the pain was originally in the diseased tooth, but it went from that to the healthy one. The explanation we need not go far to find The current of irritation travels from the carious tooth along the dental nerve to its ganglionic cell, and this under the painful excitement becomes flushed and hyperæmic. Now this excessive hyperæmia extends and overflows into the adjacent grey cell which happens to belong to the healthy tooth. The result is, that the urgent pain in the carious tooth is relieved, and the healthy one aches because its centre is hyperæmic. Beware, then, of extracting that sound tooth. If, after an operation, you find the tooth in an incurable state, your satisfaction must be complete. If, on the other hand, the tooth is a healthy one, and its extraction unnecessary, what qualms your conscience must suffer. Your success in life depends on your reputation, and your reputation on your professional skill. Convince yourselves first, as far as possible, that a tooth is in an irremediable condition, ere you extract it; and I say this, if the neuralgia be severe, and the tooth appears to be the cause. A very small amount of caries may, in a neurotic patient, give rise to extreme pain, but a soothing dressing and a suitable stopping, accompanied by some quinine or arsenic internally, cures your case. These drugs are tonics to the nerves and to the blood, and they promote the nutrition of the body, hence their marvellous effects in some cases of neuralgia. I hope I am not irrelevant in saying that the Styrians use arsenic to improve their horses' coats. Teeth are epidermal tissues as well as horses' coats.

Phosphorus is essentially a fat forming medicine. Now you will remember that the middle coat of a nerve—the white substance of Schwann—is almost entirely composed of a fatty menstrum. Therefore phosphorus, and among the rest milk, cream, butter and bacon, are of eminent service in the neuralgia of wasting nerves. For an exhausting neuralgia of some weeks' duration, a dose of gr. xx of bromide of potassium with gr. x of chloral every two hours, is very effective. But those sedatives should never be a substitute for the dental surgeon; and, moreover, they ought always to be followed up by some nerve tonic, such as cod liver oil.

Croton chloral has been in use now for more than fifteen years, and there are cases of tic that are best treated by this remedy. It exercises a selective action on the 5th nerve and affords relief without any unpleasant after effects. But in the true epileptiform neuralgia of Trousseau the best treatment undoubtedly is to adminster that "sweet oblivious antidote," chloroform, and then inject morphia hypodermically. This variety of pain is not associated with dental lesions as a rule; therefore it would be an unhappy course to extract teeth for the cure thereof. The treatment of chloroform inhalation, followed by a hypodermic injection of morphia afterwards, is, singularly enough, the orthodox treatment for spasms of internal muscles, and for delirium of some kinds. So that a very pretty algebraical picture is thrust upon our minds, full of interest, truth and suggestiveness. It is this—

| | | |
|---|---|---|
| Neuralgia : a nerve | : : Spasm | : a muscle |
| | : : Delirium | : the mind |

and we can go further and say—

| | | |
|---|---|---|
| Numbness : a nerve | : : Paralysis | : a muscle |
| | : : Coma | : the mind |

In a case of sick headache, technically known as migraine, the storm is sometimes preceded by neuralgia of one auriculo-temporal nerve. If aconite be applied externally over the painful area, and extract of Indian hemp administered internally in 1-gr. doses every 2 or 3 hours, relief will come safely and quickly. But disabuse your minds of the hope of curing a case of migraine by simply extracting a tooth. It is a possible but a very unlikely result. Iron is an exceedingly valuable medicine when the patient's gums are pale, whatever the lesions in the teeth may be. If the neuralgia be due to inflammation, no medicine is better than antimonial wine in xv. m. does every hour.

If there be periostitis, a brisk counter-irritant should be applied to the gum in the neighbourhood. A good liniment for this purpose is made by mixing equal parts of Fleming's tincture of aconite and a strong liniment of iodine (iodine the strength of the Pharmacopœia liniment). In all cases of neuralgia it is your paramount duty to examine the teeth very carefully and then decide on your treatment.

If we could take a leaf out of many past lives it would astonish us to know how many teeth have been extracted that might have been saved if a little more care and attention had been bestowed. For, after all, the practice of your profession is not summed up in the words "Operate as much as you can," but rather in doing your utmost to preserve the teeth of your patients, and save them from as much future discomfort as possible. And the value of a single tooth must not be forgotten. My paper would be sadly incomplete if I did not express my sentiments on the other side of the question I have been discussing from its conservative aspect. I must tell you I have a very high appreciation of your profession, and of the noble position you may reach by patient earnest work. The day will come with its golden opportunity (and may it come often!) when you will strike with your forceps well home round a sinful tooth, wrench it from its socket, and so save your patient from a lifelong misery, from epileptiform seizures, or from a state truly deplorable, yet none the less real— a state sans eyes, sans ears, sans taste, sans everything. Neither the dentist nor the doctor can afford to dispense with each other in this cosmopolitan problem—the treatment of pain. The dentist does his work admirably and precisely, but it requires a prescription from the doctor to restore the nerves to the normal state they were in before they contracted the evil habit of aching. The physician who treats a facial neuralgia without consulting a dental surgeon drags his patient, as a rule, into a slough of despond, and himself into professional disrepute. You have no doubt observed that I have omitted many remedies of undoubted efficacy in the treatment of pain, notably stretching and excision of a nerve, and the application of the electric current. I could not say all I would have wished to say, but if I have warned you against too hurried a diagnosis, if I have encouraged you to forget crooked creeds and worn-out dogmas respecting the treatment of pain; if, in short, I have led you into an atmosphere of more thoughtful and conscientious work, I shall be glad to know your sympathies are mine, and each of us can say with Matthew Arnold, in his "Obermann," that—

> ". . . . rigorous teachers trained my youth,
> And purged its faith and cleansed its fire;
> Showed me the high white star of truth,
> There bade me gaze and there aspire."

—*London Dental Record.*

